# Behavioral and Cortical Effects during Attention Driven Brain-Computer Interface Operations in Spatial Neglect: A Feasibility Case Study

**DOI:** 10.3389/fnhum.2017.00336

**Published:** 2017-06-28

**Authors:** Luca Tonin, Marco Pitteri, Robert Leeb, Huaijian Zhang, Emanuele Menegatti, Francesco Piccione, José del R. Millán

**Affiliations:** ^1^Chair in Brain-Machine Interface, Center for Neuroprosthetics, École Polytechnique Fédérale de LausanneGeneva, Switzerland; ^2^Neurology Section, Department of Neurosciences, Biomedicine and Movement Sciences, University of VeronaVerona, Italy; ^3^Intelligent Autonomous Systems Laboratory, Department of Information Engineering, University of PadovaPadova, Italy; ^4^Laboratory of Neuropsychology, IRCCS San Camillo Hospital FoundationVenice, Italy; ^5^Laboratory of Neurophysiology, IRCCS San Camillo Hospital FoundationVenice, Italy

**Keywords:** brain-computer interface, spatial neglect, covert visuospatial attention, electroencephalogram, alpha oscillations, functional connectivity

## Abstract

During the last years, several studies have suggested that Brain-Computer Interface (BCI) can play a critical role in the field of motor rehabilitation. In this case report, we aim to investigate the feasibility of a covert visuospatial attention (CVSA) driven BCI in three patients with left spatial neglect (SN). We hypothesize that such a BCI is able to detect attention task-specific brain patterns in SN patients and can induce significant changes in their abnormal cortical activity (α-power modulation, feature recruitment, and connectivity). The three patients were asked to control online a CVSA BCI by focusing their attention at different spatial locations, including their neglected (left) space. As primary outcome, results show a significant improvement of the reaction time in the neglected space between calibration and online modalities (*p* < 0.01) for the two out of three patients that had the slowest initial behavioral response. Such an evolution of reaction time negatively correlates (*p* < 0.05) with an increment of the Individual α-Power computed in the pre-cue interval. Furthermore, all patients exhibited a significant reduction of the inter-hemispheric imbalance (*p* < 0.05) over time in the parieto-occipital regions. Finally, analysis on the inter-hemispheric functional connectivity suggests an increment across modalities for regions in the affected (right) hemisphere and decrement for those in the healthy. Although preliminary, this feasibility study suggests a possible role of BCI in the therapeutic treatment of lateralized, attention-based visuospatial deficits.

## Introduction

Spatial neglect (SN) is one of the most frequent and disabling neuropsychological syndromes following right-hemisphere damage (Heilman et al., 2003; Buxbaum et al., 2004; Adair and Barrett, 2008). SN patients usually fail to report stimuli in the contralesional side of space, rendering difficult their effective perception of the surrounding space. Although some spontaneous recovery occurs in the majority of patients after stroke, SN might remain severe in the chronic phase, limiting considerably the effectiveness of rehabilitation interventions (Katz et al., 1999; Battelli et al., 2001; Jehkonen et al., 2006; Kerkhoff and Schenk, 2012; Bowen et al., 2013; Riestra and Barrett, 2013).

One of the most accredited hypothesis explaining SN was introduced by Kinsbourne and it relies on the concept of inter-hemispheric rivalry (Kinsbourne, 1993). In terms of attentional vectors, it is assumed that lesions to the right-hemisphere provoke changes in neural activation inducing inter-hemispheric imbalance and, as a consequence, a hypoexploration of the left (neglected) space and a hyperattention toward the right.

Recently, it has been proposed that SN should be better attributed to abnormalities in the functional organization of large fronto-parietal attention networks, rather than lesions in local cerebral areas (Corbetta et al., 2005; He et al., 2007; Bartolomeo et al., 2012). Furthermore, it has been demonstrated that the normalization of the inter-hemispheric connectivity is a physiological signature of recovery from SN (Baldassarre et al., 2014; Ramsey et al., 2016).

Behavioral treatments of SN are heterogeneous, because they are based on different theoretical concepts (Riestra and Barrett, 2013). Most of them, however, are based on the concept of re-orienting the visuospatial attention toward the neglected side of space (Kortte and Hillis, 2011; Priftis et al., 2013; Azouvi et al., 2016). Recently, it has been demonstrated that such interventions may have direct effects in the neural mechanisms of SN patients (Saj et al., 2013). Furthermore, novel approaches have been proposed based on neurofeedback or brain stimulation techniques in order to suppress/enhance the hyper/hypo-activity in the healthy/affected hemisphere (Kortte and Hillis, 2011; Müri et al., 2013; Okazaki et al., 2014; Robineau et al., 2014).

The current study aims at evaluating the feasibility of a novel approach based on Brain-Computer Interface (BCI). The last years have seen a growing interest of the scientific community in identifying new directions and different target populations for BCI-driven control and rehabilitation (Brunner et al., 2015). Indeed, beyond the use as an assistive device (Birbaumer, 2006; Sellers et al., 2014; Leeb et al., 2015), evidences suggest that BCI can play a relevant role in motor rehabilitation by associating actual or imagined motor tasks to a coherent, real-time feedback provided to the patient (Daly and Wolpaw, 2008; Grosse-Wentrup et al., 2011; Ramos-Murguialday et al., 2013; Ang and Guan, 2017). Focusing on electroencephalography (EEG)-based studies, recent reviews have highlighted the potential benefits of BCI approaches in motor rehabilitation after stroke (Monge-Pereira et al., 2017; Remsik et al., 2017). Based on the same principles, we hypothesized that BCI systems might also be adopted in the case of cognitive rehabilitation of SN. Our BCI approach exploits the covert visuospatial attention (CVSA) orienting paradigm (Tonin et al., 2012; Marchetti et al., 2013; Tonin et al., 2013) that might offer a direct backdoor to the impaired visuospatial attention mechanisms of SN patients.

Herein, we report the cases of three SN patients who operated an EEG-based CVSA BCI during two consecutive weeks. We evaluated the ability of each patient to operate the BCI and we analyzed possible neurophysiological changes during online BCI operations. To the best of our knowledge, this is the first attempt of using a CVSA BCI and analyzing its contingency effects in SN patients.

## Case Description

Three SN patients (P1-3, from 46 to 61 years old, median 57; two females) with unilateral right-hemisphere damage participated in the study. Patients did not have previous experience with BCI systems. Written informed consent was obtained from all patients to participate in the study, to collect data and to publish information appearing in this case report. Patients were tested in accordance with the Helsinki declaration. The study was approved by the institutional ethical committee (*Nucleo per la Ricerca Clinica*) of the IRCCS San Camillo Hospital Foundation, Venice, Italy. All patients had unilateral lesions because of first stroke (time from lesion: 4, 8, and 13 months, respectively; Supplementary Table 1). Lesion sites were confirmed by magnetic resonance imaging scans and localized in the fronto-parietal lobe and in the insula (P1); in the capsulo-thalamic area and in the insula (P2); and in the fronto-temporal-parietal lobe (P3).

Patients were assessed with a screening test to exclude general cognitive impairment (Measso et al., 1993), and with a battery of neuropsychological tests to detect SN in the peripersonal space (Wilson et al., 1987; Vallar et al., 1994). Clinical signs of SN were present in each patient (P1: BIT-conventional = 104, cut-off < 130; P2: MMSE = 23.31, cut-off < 24; BIT-conventional = 122, cut-off < 130; Symbol cancelation: 3, -3 ≤ cut-off ≥ 3; P3: Symbol cancelation = 5, -3 ≤ cut-off ≥ 3; Supplementary Table 2).

## Materials and Methods

### Experimental Design

**FIGURE 1:**
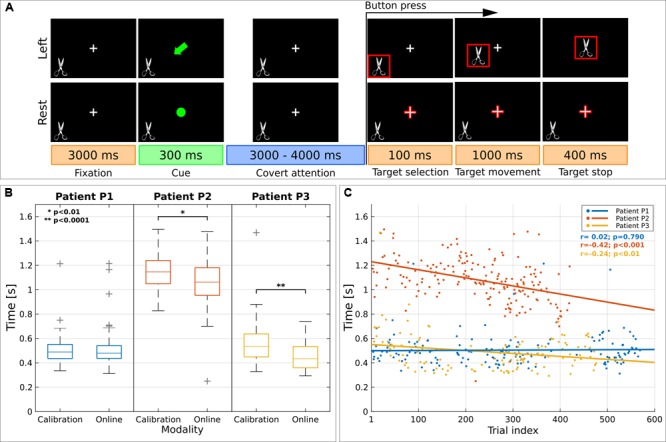
Visual paradigm and reaction time (RT). **(A)** Schematic representation of the paradigm presented to spatial neglect (SN) patients. Each trial started with a fixation period (3000 ms) where patients had to gaze at a cross, visible at the center of the screen. Then the cross was replaced by a symbolic cue (300 ms) indicating the to-be-attended location. The covert attention period lasted for 3000–4000 ms; afterward, one of the two to-be-attended locations was highlighted in red (target selection, 100 ms) as online feedback of the classification result. Immediately afterward—in the case of left target—the image started moving toward the center of the screen (target movement, 1000 ms) and it disappeared after 400 ms (target stop). SN patients were required to press a button with their right hand as soon as they perceived the images highlighted in red. **(B)** Analysis on RT for left covert visuospatial attention (CVSA) task. Distribution of RT for each patient across modality. For each box, median is reported. Box edges represent the 25th and 75th percentile of Individual α-Power (IAP). Student t-test outcomes are annotated in each plot. **(C)** Scatter plot shows the evolution of RT over time. Results are reported for each patient (in blue, red, and yellow for P1, P2 and P3, respectively). Lines represent the least-squares fit to the scatter plot for each patient. Spearman’s correlation and significance are annotated.

Patients were asked to control an online two-class BCI by means of a voluntary CVSA-orienting task (**Figure 1A**). Each trial started with a fixation cross (size 3.12°) in the middle of the screen and a random image of real object (4.8°) at the bottom left (neglected side). After 3000 ms, patients were instructed by a cue (300 ms) to covertly focus their attention to the left target (attention condition) or to keep fixating the center of the screen (rest condition). After a random time (3000–4000 ms), the target was outlined in red. In case of left target selection, the image started moving toward the center. Patients were required to press a button with their right hand as soon as they perceived the target selection.

Each patient performed six recording sessions within two consecutive weeks. In average, each session consisted of 2.7 ± 0.8 runs and each run of 30 trials randomly shuffled between attention (20) and rest (10) condition (Supplementary Table 3). The first two sessions were devoted to the calibration of the BCI with a positive feedback always delivered to patients; in the following runs (online modality) the image selected at the end of the trial was based on the output of the CVSA BCI, as real-time feedback for the patients.

### CVSA BCI

Electroencephalography signals were acquired with a 64-channel system at 2048 Hz (BioSemi, Amsterdam, Netherlands). Electrodes were placed according to the standard international 10–20 system. Eye movements were recorded by means of three electrodes placed at the outer canthi of the eyes and at the gabella. The CVSA BCI was similar to our previous work (Tonin et al., 2012, 2013). The envelope of the EEG was extracted in seven α sub-bands (8–14 Hz, with 3 Hz of bandwidth) by means of Hilbert transform and a Laplacian filter was applied. Channels were pre-selected in the parieto-occipital regions (17 electrodes: P7–8, PO7–8, O1–2). Trial classification was based on data from the first 3000 ms after the cue. This period was split into windows of 150 ms. For each window, a quadratic discriminant analysis classifier was trained with the most discriminant features (frequency-channel pairs) selected during the calibration. In the online modality, classifiers were evaluated and the resulting posterior probabilities were integrated over time to deliver the final decision at the end of the trial.

### Neurophysiological Analysis

#### Data Processing

Data were spatially filtered with Common Average Reference and periodogram was extracted in the 4–48 Hz frequency range (1 Hz resolution, 0.0625 ms shift, 1000 ms window). Trials were extracted in the pre-cue interval. We defined six nodes (frontal, parietal, and occipital regions for the left- and right-hemisphere; Supplementary Table 4) according to (Baldassarre et al., 2014).

#### Individual α-Frequency (IAF) and Individual α-Power (IAP)

We selected symmetric nodes in the parieto-occipital regions (Thut et al., 2006). The spectrum of each channel was normalized for inter-trial comparisons. For each hemisphere, the IAF was defined as the first peak in the frequency range between 6 and 12 Hz (Supplementary Table 5). If no peak was found in the right (affected) hemisphere, the corresponding average peak computed in the same modality was selected. Therefore, Individual α-Power (IAP) was computed as the average around IAF (±1 Hz) for each trial and for each hemisphere.

#### Inter-Hemispheric Discriminancy

First, we selected those features that patients were modulating during BCI operations. Second, we extracted those related to the IAF of each patient. Third, for each run we computed the Fisher Score (FS) values of each feature as follows:

$$\begin{array}{c} FS_{k}=\frac{\left|\mu_{k}^{ATTENTION}-\mu_{k}^{REST}\right|}{\sqrt{\sigma_{k}^{ATTENTION}+\sigma_{k}^{REST}}} \end{array}$$

where *k* indicates the k^th^ feature, *μ* the mean and *σ* the variance over the attention and rest conditions. This metric represents the participants’ ability to modulate their neural networks accordingly to the CVSA-orienting task. Fourth, for each run we computed the differences between the FS of each homotopic nodes (right minus left hemisphere).

#### Inter-Hemispheric Functional Connectivity (FC)

Analysis on FC was based on (Baldassarre et al., 2014). First, for each patient, IAP was extracted in the trial interval (left CVSA task). Second, we averaged the IAP of the channels belonging to each aforementioned nodes. Third, we generated a channel-wise FC map for each node by extracting the time-course of the IAP for the node and computing the Pearson’s correlation coefficient between that time-course and the time-course of all the other channels. Finally, we computed the inter-hemispheric connectivity for each node by averaging the FC values of those channels belonging to the nodes in the opposite hemisphere.

#### Statistical Tests

Statistics were based on Student’s *t*-test between data distributions from the two modalities (calibration vs. online). Statistical significance values reported in the paper are Bonferroni corrected.

## Results and Discussion

### CVSA BCI Online Accuracy

We computed BCI performance as the percentage of trials that the BCI classified correctly during each online session. The average performance was 55 ± 3.9%, 60 ± 2.8% and 58.3 ± 3.7% (median and standard error) for patients P1, P2, and P3. Although patients’ BCI performance was low, it was above random for most sessions with an individual maximum accuracy of 76.6, 70, and 70% in their best runs. Furthermore, such a level of BCI accuracy is similar to that achieved by stroke patients during BCI-based motor rehabilitation (Prasad et al., 2010; Ramos-Murguialday et al., 2013; Pichiorri et al., 2015).

A posteriori analysis of ocular artifacts showed that horizontal eye movements were moderate for all three patients (9.4, 3.6, and 25% of the trials for P1, P2, and P3). Furthermore, the mean square contingency coefficient between the direction of horizontal eye movements and the CVSA tasks was not significant (P1: φ^2^ = 0.53, *p* = 0.46; P2: φ^2^= 0.83, *p* = 0.36; P3: φ^2^= 0.29, *p* = 0.58).

### Behavioral Outcome

**Figures 1B,C** report the analysis of the behavioral response to the button press across modalities (calibration or online) and over time for left CVSA task. Results showed a decrement of the reaction time (RT) for patients P2 and P3 (1.15 ± 0.15 s vs. 1.06 ± 0.17 s, *p* < 0.01 and 0.57 ± 0.20 s vs. 0.45 ± 0.10 s, *p* < 0.001). Patient P1 did not exhibit any change in RTs (0.50 ± 0.13 s vs. 0.50 ± 0.13 s; *p* = 0.72), however, he had the fastest initial RTs. A significant negative correlation between RTs and trial index was found for patients P2 and P3 (*r* = -0.42, *p* < 0.001 and *r* = -0.24, *p* < 0.01). The speedup in RTs cannot be explained as a consequence of behavioral training, since the duration of the trials was random.

### Inter-Hemispheric Asymmetry of IAF and IAP

We analyzed the spectral and spatial locations of the IAF in the parieto-occipital regions during the pre-cue interval for each patient, modality and hemisphere. During calibration the percentage of trials with missing α-peak was significantly higher in the affected hemisphere (1.73 ± 2.71% vs. 10.02 ± 7.68%, *p* < 0.01; mean and standard deviation for healthy vs. affected hemisphere). However, during the online modality, a reduction of this percentage seems to occur (1.73 ± 3.1% vs. 7.52 ± 7.90%, *p* < 0.01). This spatial asymmetry substantially differs from healthy populations, where a symmetric α-response is expected along the two hemispheres (Klimesch et al., 1998; de Munck et al., 2007).

**FIGURE 2:**
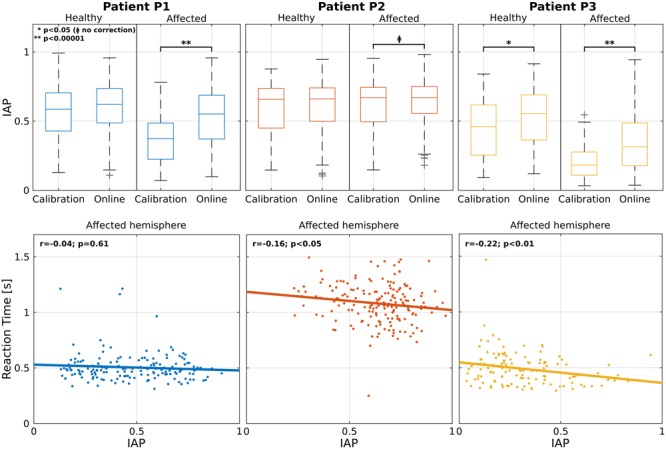
Analysis on the IAP. Individual patient results are grouped by column. First row shows the IAP averaged across calibration and online modalities for healthy (left) and affected (right) parieto-occipital nodes. For each box, median is reported. Box edges represent the 25th and 75th percentile of IAP. Student *t*-test outcomes are annotated in each plot. In the second row, the scatter plots show the relation between IAP and RT for the parieto-occipital nodes in the affected (right) hemisphere. Each point corresponds to a trial. Lines represent the least-squares fit to the scatter plot. Spearman’s correlation and significance are annotated in each plot.

A comparison of the related IAP is reported in **Figure 2** (first row) for each modality and hemisphere. Statistical analysis showed an increment in the affected hemisphere for all patients during the online modality (statistically significant for P1 and P3: *p* < 0.00001; marginally significant for P2: *p* < 0.05 without correction). For patient P3 the increment was significant also in the healthy hemisphere.

Furthermore, the aforementioned restoration of the α-peak in the pre-cue interval seems to anticipate the decrement of the RTs in the correctly classified left trials (**Figure 2**, second row). In fact, patients P2 and P3 showed a significant negative correlation between the RTs and the IAP in the affected hemisphere (*r* = -016, *p* < 0.05 and *r* = -0.22, *p* < 0.01). This did not apply for patient P1 (*r* = -0.04, *p* = 0.61) but he/she was the only one who showed constantly low RTs over all runs. These results are in line with the literature, where it has been demonstrated that exists a positive correlation between the strength of α-power in the pre-stimulus interval and a faster RT (Klimesch et al., 1996; Nenert et al., 2012).

### Inter-Hemispheric Asymmetry of Features Discriminancy

Feature discriminancy is a common BCI metric to assess the subject’s ability of modulating channel-frequency pairs during different mental tasks (Galán et al., 2007; Leeb et al., 2015). Herein, we investigated possible inter-hemispheric changes in the spatial distribution of discriminancy across the two experimental modalities (calibration and online).

**FIGURE 3:**
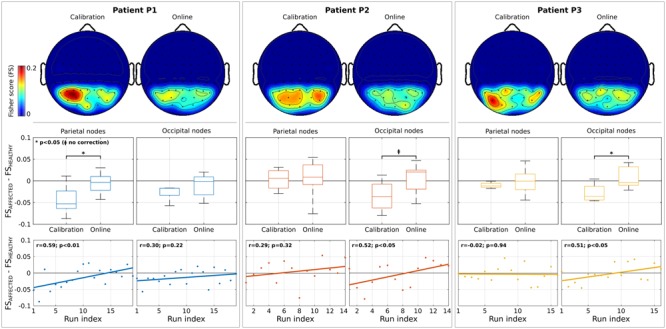
Analysis on features discriminancy. Individual patient results are grouped by column. First row illustrates the topographic maps of Fisher Score (FS) values normalized between 0 and 1 (for comparison purposes) for calibration and online modality. Low and high discriminancy is reported in blue and red, respectively. Only the parieto-occipital channels used as input for Brain-Computer Interface (BCI) online operations are shown. Second row depicts the difference of FS values between nodes belonging to affected and healthy hemisphere (parieto and occipital regions, separately) for each modality. In each box, median is reported. Box edges represent the 25th and 75th of FS. Negative values correspond to a stronger modulation in the healthy (right) hemisphere. Student *t*-test outcomes are annotated in each plot. Third row reports the evolution over runs of the difference of FS values. Each point corresponds to the value computed in a run. Lines represent the least-square fit to the scatter plot. Spearman’s correlation and significance are annotated in each plot.

Topographic maps in **Figure 3** show the spatial distribution of discriminancy during calibration and online modalities (only parieto-occipital channels exploited in the online BCI are reported; normalized values are shown for comparison purposes; low discriminancy in blue, high in red). An initial imbalance of the discriminancy toward the healthy hemisphere (patients P1 and P3) and toward the affected one (patient P2) seems to be attenuated in the online modality. This hypothesis is supported by analysis on the difference of discriminancy between homotopic regions (**Figure 3**, second row). Negative values correspond to asymmetry in the modulation toward the left (healthy) hemisphere. A general asymmetry reduction (values toward zero) was reported for all patients in the parietal or the occipital regions with statistical significance for P1 and P3 (*p* < 0.05) and marginal significance for P2 (*p* < 0.05, without correction). Furthermore, for all patients, the asymmetry toward the healthy hemisphere significantly decreased across runs (P1, parietal regions: *r* = 0.59, *p* < 0.01; P2 and P3, occipital regions: *r* = 0.52, *p* < 0.05 and *r* = 0.51, *p* < 0.05) (**Figure 3**, third row).

Inter-patient differences might be explained by the fact that the most discriminant channels are strictly subject-dependent during CVSA tasks, as already reported in literature (Tonin et al., 2012, 2013). These results are in line with the rivalry hypothesis of SN (Kinsbourne, 1993) with the additional advantage of contingency with respect to the attention task performed by the patients.

### Inter-Hemispheric FC

**FIGURE 4:**
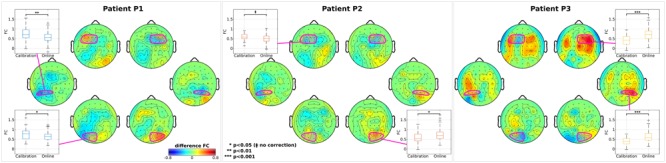
Analysis on inter-hemispheric connectivity. For each patient, topographic maps of z-transformed functional connectivity (FC) between each node and the whole opposite hemisphere are reported. Difference between online and calibration modality is reported for visualization purposes. In each map, nodes (and channels belonging to) are highlighted in magenta. Box plots represent the distributions of the z-transformed FC in the calibration and online modality. Only nodes with statistical significance difference between modality are shown. In each box, median is reported. Box edges represent the 25th and 75th of the z-transformed FC. Student *t*-test outcomes are annotated in each plot.

Several functional Magnetic Resonance Imaging (fMRI) studies reported a relation between FC in large scale resting-state networks and SN (Corbetta et al., 2005; He et al., 2007). On the other hand, our within-patient connectivity analyses focused on investigating the FC during the attention task (driven by the CVSA BCI) and possible changes between calibration and online trials by exploiting the high spectro-temporal resolution of EEG signals. A general inter-patient trend seems to appear, highlighting an increment of inter-hemispheric FC for nodes in the affected (right) hemisphere and decrement for those in the healthy (left) one (**Figure 4**). Topographic plots show the difference between online and calibration FC maps computed with respect to each of the selected nodes (electrodes highlighted in magenta). Boxplots illustrate the difference in calibration and online modality between the FC of each node and the FC averaged across the whole opposite hemisphere. Only statistically significant differences are reported. For patient P2 the inter-hemispheric FC increment happened in the right-occipital node (*p* < 0.05) and for patient P3 in right-frontal (*p* < 0.001) and right-parietal nodes (*p* < 0.001). Contrarily, for patient P1, decrement of FC occurred in left-parietal (*p* < 0.01) and left-occipital nodes (*p* < 0.05) and, again, for patient P2 marginally in left-frontal node (*p* < 0.05, without correction). Changes in FC did not correlate with RT for any patient or node. Previous studies in resting-state networks demonstrated that the increasing inter-hemispheric connectivity (from right parietal regions to left hemisphere) is a signature of recovery from SN (Ramsey et al., 2016). The missing correlation between connectivity changes and RT might be due to the task-dependent nature of our analysis and, consequently, to the task-induced specific correlation patterns between different cortical regions (Zanto et al., 2011) as already suggested in (Baldassarre et al., 2014).

## Concluding Remarks

To the best of our knowledge, for the first time cortical effects in EEG patterns have been monitored during online CVSA BCI operations performed by SN patients. As primary outcome, our study showed that three patients suffering from SN can actively control a CVSA BCI. Nonetheless, an improvement of such a level of control might be desirable to enhance the neurofeedback- driven reward and, as a consequence, the neuroplasticity response (Kaiser et al., 2012; Young et al., 2014). In this regard, additional neural correlates of CVSA [e.g., the lateralization index (Thut et al., 2006)] and new training paradigms can be investigated in future studies. From the neurophysiological point of view, inter-modality analyses on the restoration of the initial α-response asymmetry, as well as on the increment of modulation and connectivity in the affected hemisphere, suggest a positive effect of the online BCI feedback. However, it should be noticed that causality between online BCI operations and changes in the inter-hemispheric activity cannot be firmly established yet, given the limited population size and the uncontrolled experimental design. Further studies are required in order to verify such hypotheses. First, it is mandatory to run a long-term randomized controlled trial with larger groups to identify the effective contribution of the online BCI feedback. Second, it is also crucial to probe with other functional techniques, such as fMRI, the actual recruitment of perilesional areas and their role in inter-hemispheric activity. Finally, such a kind of CVSA BCI should be combined with tailored cognitive interventions to optimize the clinical outcomes and to reduce the confounding factors and the within-patient variability effects.

## Author Contributions

LT, RL, and JdRM were responsible for the study conception; LT and MP implemented and executed the experiments; LT, MP, RL, HZ, EM, FP, and JdRM contributed to the methodology, data analysis, and manuscript preparation.

## Conflict of Interest Statement

The authors declare that the research was conducted in the absence of any commercial or financial relationships that could be construed as a potential conflict of interest.

## References

[B1] Adair J. C. Barrett A. M. (2008). Spatial neglect: clinical and neuroscience review: a wealth of information on the poverty of spatial attention. *Ann. N. Y. Acad. Sci.* 1142 21–43. 10.1196/annals.1444.008 18990119 PMC2962986

[B2] Ang K. K. Guan C. (2017). EEG-based strategies to detect motor imagery for control and rehabilitation. *IEEE Trans. Neural Syst. Rehabil. Eng.* 25 392–401. 10.1109/TNSRE.2016.2646763 28055887

[B3] Azouvi P. Jacquin-Courtois S. Luauté J. (2016). Rehabilitation of unilateral neglect: evidence-based medicine. *Ann. Phys. Rehabil. Med.* 10.1016/j.rehab.2016.10.006 [Epub ahead of print]. 27986428

[B4] Baldassarre A. Ramsey L. Hacker C. L. Callejas A. Astafiev S. V. Metcalf N. V. (2014). Large-scale changes in network interactions as a physiological signature of spatial neglect. *Brain* 137 3267–3283. 10.1093/brain/awu297 25367028 PMC4240302

[B5] Bartolomeo P. Thiebaut de Schotten M. Chica A. B. (2012). Brain networks of visuospatial attention and their disruption in visual neglect. *Front. Hum. Neurosci.* 6:110 10.3389/fnhum.2012.00110 PMC3343690 22586384

[B6] Battelli L. Cavanagh P. Intriligator J. Tramo M. J. Hénaff M. A. Michèl F. (2001). Unilateral right parietal damage leads to bilateral deficit for high-level motion. *Neuron* 32 985–995. 10.1016/S0896-6273(01)00536-0 11754832

[B7] Birbaumer N. (2006). Breaking the silence: brain-computer interfaces (BCI) for communication and motor control. *Psychophysiology* 43 517–532. 10.1111/j.1469-8986.2006.00456.x 17076808

[B8] Bowen A. Hazelton C. Pollock A. Lincoln N. B. (2013). Cognitive rehabilitation for spatial neglect following stroke. *Cochrane Database Syst. Rev.* 7:CD003586 10.1002/14651858.CD003586.pub3 PMC6464849 23813503

[B9] Brunner C. Birbaumer N. Blankertz B. Guger C. Kübler A. Mattia D. (2015). BNCI horizon 2020: towards a roadmap for the BCI community. *Brain Comput. Interfaces* 2 1–10. 10.1080/2326263X.2015.1008956

[B10] Buxbaum L. J. Ferraro M. K. Veramonti T. Farne A. Whyte J. Ladavas E. (2004). Hemispatial neglect: subtypes, neuroanatomy, and disability. *Neurology* 62 749–756. 10.1212/01.WNL.0000113730.73031.F4 15007125

[B11] Corbetta M. Kincade M. J. Lewis C. Snyder A. Z. Sapir A. (2005). Neural basis and recovery of spatial attention deficits in spatial neglect. *Nat. Neurosci.* 8 1603–1610. 10.1038/nn1574 16234807

[B12] Daly J. J. Wolpaw J. R. (2008). Brain-computer interfaces in neurological rehabilitation. *Lancet Neurol.* 7 1032–1043. 10.1016/S1474-4422(08)70223-0 18835541

[B13] de Munck J. C. Gonçalves S. I. Huijboom L. Kuijer J. P. Pouwels P. J. Heethaar R. M. (2007). The hemodynamic response of the alpha rhythm: an EEG/fMRI study. *Neuroimage* 35 1142–1151. 10.1016/j.neuroimage.2007.01.022 17336548

[B14] Galán F. Ferrez P. W. Oliva F. Guardia J. Millán J. d. R. (2007). “Feature extraction for multi-class BCI using canonical variates analysis,” in *Proceedings of the IEEE International Symposium on Intelligent Signal Processing* Alcalá de Henares 1–6. 10.1109/WISP.2007.4447615

[B15] Grosse-Wentrup M. Mattia D. Oweiss K. (2011). Using brain-computer interfaces to induce neural plasticity and restore function. *J. Neural Eng.* 8:25004 10.1088/1741-2560/8/2/025004 PMC4515347 21436534

[B16] He B. J. Snyder A. Z. Vincent J. L. Epstein A. Shulman G. L. Corbetta M. (2007). Breakdown of functional connectivity in frontoparietal networks underlies behavioral deficits in spatial neglect. *Neuron* 53 905–918. 10.1016/j.neuron.2007.02.013 17359924

[B17] Heilman K. M. Watson R. T. Valenstein E. (2003). “Neglect and related disorders,” in *Clinical Neuropsychology* 4th Edn eds Heilman K. M. Valenstein E. (Oxford: Oxford University Press) 243–293.

[B18] Jehkonen M. Laihosalo M. Kettunen J. E. (2006). Impact of neglect on functional outcome after stroke: a review of methodological issues and recent research findings. *Restor. Neurol. Neurosci.* 24 209–215. 17119299

[B19] Kaiser V. Daly I. Pichiorri F. Mattia D. Müller-Putz G. R. Neuper C. (2012). Relationship between electrical brain responses to motor imagery and motor impairment in stroke. *Stroke* 43 2735–2740. 10.1161/STROKEAHA.112.665489 22895995

[B20] Katz N. Hartman-Maeir A. Ring H. Soroker N. (1999). Functional disability and rehabilitation outcome in right hemisphere damaged patients with and without unilateral spatial neglect. *Arch. Phys. Med. Rehabil.* 80 379–384. 10.1016/S0003-9993(99)90273-3 10206598

[B21] Kerkhoff G. Schenk T. (2012). Rehabilitation of neglect: an update. *Neuropsychologia* 50 1072–1079. 10.1016/j.neuropsychologia.2012.01.024 22306520

[B22] Kinsbourne M. (1993). “Orientational bias model of unilateral neglect: evidence from attentional gradients within hemispace,” in *Unilateral Neglect: Clinical and Experimental Studies (Brain Damage, Behaviour and Cognition)* eds Marshall J. Robertson I. (Hove: Psychology Press) 63–86.

[B23] Klimesch W. Doppelmayr M. Russegger H. Pachinger T. Schwaiger J. (1998). Induced alpha band power changes in the human EEG and attention. *Neurosci. Lett.* 244 73–76. 9572588 10.1016/s0304-3940(98)00122-0

[B24] Klimesch W. Doppelmayr M. Schimke H. Pachinger T. (1996). Alpha frequency, reaction time, and the speed of processing information. *J. Clin. Neurophysiol.* 13 511–518. 8978623 10.1097/00004691-199611000-00006

[B25] Kortte K. B. Hillis A. E. (2011). Recent trends in rehabilitation interventions for visual neglect and anosognosia for hemiplegia following right hemisphere stroke. *Fut. Neurol.* 6 33–43. 10.2217/fnl.10.79 PMC3039433 21339836

[B26] Leeb R. Tonin L. Rohm M. Desideri L. Carlson T. Millan J. d. R. (2015). Towards independence: a BCI telepresence robot for people with severe motor disabilities. *Proc. IEEE* 103 969–982. 10.1109/JPROC.2015.2419736

[B27] Marchetti M. Piccione F. Silvoni S. Gamberini L. Priftis K. (2013). Covert visuospatial attention orienting in a brain-computer interface for amyotrophic lateral sclerosis patients. *Neurorehabil. Neural Repair* 27 430–438. 10.1177/1545968312471903 23353184

[B28] Measso G. Cavarzeran F. Zappalà G. Lebowitz B. D. Crook T. H. Pirozzolo F. J. (1993). The mini-mental state examination: normative study of an Italian random sample. *Dev. Neuropsychol.* 9 77–85. 10.1080/87565649109540545

[B29] Monge-Pereira E. Ibañez-Pereda J. Alguacil-Diego I. M. Serrano J. I. Spottorno-Rubio M. P. Molina-Rueda F. (2017). Use of electroencephalography brain computer interface systems as a rehabilitative approach for upper limb function after a stroke. A systematic review. *Am. Acad. Phys. Med. Rehabil.* 10.1016/j.pmrj.2017.04.016 [Epub ahead of print]. 28512066

[B30] Müri R. M. Cazzoli D. Nef T. Mosimann U. P. Hopfner S. Nyffeler T. (2013). Non-invasive brain stimulation in neglect rehabilitation: an update. *Front. Hum. Neurosci.* 7:248 10.3389/fnhum.2013.00248 PMC3677145 23772209

[B31] Nenert R. Viswanathan S. Dubuc D. M. Visscher K. M. (2012). Modulations of ongoing alpha oscillations predict successful short-term visual memory encoding. *Front. Hum. Neurosci.* 6:127 10.3389/fnhum.2012.00127 PMC3347628 22586390

[B32] Okazaki Y. O. Horschig J. M. Luther L. Oostenveld R. Murakami I. Jensen O. (2014). Real-time MEG neurofeedback training of posterior alpha activity modulates subsequent visual detection performance. *Neuroimage* 107 323–332. 10.1016/j.neuroimage.2014.12.014 25514519

[B33] Pichiorri F. Morone G. Petti M. Toppi J. Pisotta I. Molinari M. (2015). Brain-computer interface boosts motor imagery practice during stroke recovery. *Ann. Neurol.* 77 851–865. 10.1002/ana.24390 25712802

[B34] Prasad G. Herman P. Coyle D. McDonough S. Crosbie J. (2010). Applying a brain-computer interface to support motor imagery practice in people with stroke for upper limb recovery: a feasibility study. *J. Neuroeng. Rehabil.* 7:60 10.1186/1743-0003-7-60 PMC3017056 21156054

[B35] Priftis K. Passarini L. Pilosio C. Meneghello F. Pitteri M. (2013). Visual scanning training, limb activation treatment, and prism adaptation for rehabilitating left neglect: who is the winner? *Front. Hum. Neurosci.* 7:360 10.3389/fnhum.2013.00360 PMC3703546 23847520

[B36] Ramos-Murguialday A. Broetz D. Rea M. Läer L. Yilmaz O. Brasil F. L. (2013). Brain-machine interface in chronic stroke rehabilitation: a controlled study. *Ann. Neurol.* 74 100–108. 10.1002/ana.23879 23494615 PMC3700597

[B37] Ramsey L. E. Siegel J. S. Baldassarre A. Metcalf N. V. Zinn K. Shulman G. L. (2016). Normalization of network connectivity in hemispatial neglect recovery. *Ann. Neurol.* 80 127–141. 10.1002/ana.24690 27277836 PMC5682026

[B38] Remsik A. Young B. Vermilyea R. Kiekoefer L. Abrams J. Elmore S. E. (2017). A review of the progression and future implications of brain-computer interface therapies for restoration of distal upper extremity motor function after stroke. *Expert Rev. Med. Devices* 13 445–454. 10.1080/17434440.2016.1174572.A PMC5131699 27112213

[B39] Riestra A. R. Barrett A. M. (2013). Rehabilitation of spatial neglect. *Handb. Clin. Neurol.* 110 347–355. 10.1016/B978-0-444-52901-5.00029-0 23312654 PMC3988490

[B40] Robineau F. Rieger S. W. Mermoud C. Pichon S. Koush Y. Van De Ville D. (2014). Self-regulation of inter-hemispheric visual cortex balance through real-time fmri neurofeedback training. *Neuroimage* 100 1–14. 10.1016/j.neuroimage.2014.05.072 24904993

[B41] Saj A. Cojan Y. Vocat R. Luauté J. Vuilleumier P. (2013). Prism Adaptation Enhances Activity of Intact Fronto-Parietal Areas in Both Hemispheres in Neglect Patients. *Cortex* 49 107–119. 10.1016/j.cortex.2011.10.009 22154751

[B42] Sellers E. W. Ryan D. B. Hauser C. K. (2014). Noninvasive brain-computer interface enables communication after brainstem stroke. *Sci. Transl. Med.* 6 257re7 10.1126/scitranslmed.3007801 PMC4711808 25298323

[B43] Thut G. Nietzel A. Brandt S. A. Pascual-Leone A. (2006). Alpha-band electroencephalographic activity over occipital cortex indexes visuospatial attention bias and predicts visual target detection. *J. Neurosci.* 26 9494–9502. 10.1523/JNEUROSCI.0875-06.2006 16971533 PMC6674607

[B44] Tonin L. Leeb R. Millán J. d. R. (2012). Time-dependent approach for single trial classification of covert visuospatial attention. *J. Neural Eng.* 9:45011 10.1088/1741-2560/9/4/045011 22832204

[B45] Tonin L. Leeb R. Sobolewski A. Millán J. d. R. (2013). An online EEG BCI based on covert visuospatial attention in absence of exogenous stimulation. *J. Neural Eng.* 10:56007 10.1088/1741-2560/10/5/056007 23918205

[B46] Vallar G. Rusconi M. L. Fontana S. Musicco M. (1994). Tre test Di esplorazione visuo-spaziale: taratura Su 212 soggetti normali. *Arch. Psicol. Neurol. Psichiatr.* 55 827–841.

[B47] Wilson B. A. Cockburn J. Halligan P. (1987). *Behavioural Inattention Test: Manual*. Bury St Edmunds: Thames Valley Test Company.

[B48] Young B. M. Nigogosyan Z. Walton L. M. Song J. Nair V. A. Grogan S. W. (2014). Changes in functional brain organization and behavioral correlations after rehabilitative therapy using a brain-computer interface. *Front. Neuroeng.* 7:26 10.3389/fneng.2014.00026 PMC4097124 25076886

[B49] Zanto T. P. Rubens M. T. Thangavel A. Gazzaley A. (2011). Causal role of the prefrontal cortex in top-down modulation of visual processing and working memory. *Nat. Neurosci.* 14 656–661. 10.1038/nn.2773 21441920 PMC3083493

