# Supplementary material for: Behavioral and Cortical Effects during Attention Driven Brain-Computer Interface Operations in Spatial Neglect: A Feasibility Case Study
Source: Front Hum Neurosci. 2017 Jun 28;11:336. doi: 10.3389/fnhum.2017.00336 (PMC5487481; doi:10.3389/fnhum.2017.00336)
Supplement: Supplementary file 1 [file Table_1.DOCX]

Supplementary Material

Behavioral and Cortical Effects during Attention Driven Brain-Computer Interface Operations in Spatial Neglect:
A Feasibility Case Study

Luca Tonin^*^, Marco Pitteri, Robert Leeb, Huaijian Zhang, Emanuele Menegatti, Francesco Piccione, José del R. Millán^*^

*** Correspondence:** Luca Tonin, [luca.tonin@epfl.ch](mailto:luca.tonin@epfl.ch)**,** José del R. Millán, [jose.millan@epfl.ch](mailto:jose.millan@epfl.ch)

# Supplementary Table 1

Table 1. Demographic and lesion data of SN patients. Assessed for eligibility: N=3. Inclusion criteria: absence of dementia, substance abuse, and psychiatric disorders; absence of visual field defects; unilateral right-hemisphere lesions because of first stroke.

| Patient | Lesion site | From lesion (months) | Handedness | Gender | Age (years) | Education (years) |
| --- | --- | --- | --- | --- | --- | --- |
| P1 | FP, insula | 4 | R | M | 61 | 8 |
| P2 | capsulo-thalamic area, insula | 8 | R | F | 57 | 16 |
| P3 | FTP | 13 | R | F | 46 | 8 |
