# Supplementary material for: Behavioral and Cortical Effects during Attention Driven Brain-Computer Interface Operations in Spatial Neglect: A Feasibility Case Study
Source: Front Hum Neurosci. 2017 Jun 28;11:336. doi: 10.3389/fnhum.2017.00336 (PMC5487481; doi:10.3389/fnhum.2017.00336)
Supplement: Supplementary file 2 [file Table_2.DOCX]

Supplementary Material

Behavioral and Cortical Effects during Attention Driven Brain-Computer Interface Operations in Spatial Neglect:
A Feasibility Case Study

Luca Tonin^*^, Marco Pitteri, Robert Leeb, Huaijian Zhang, Emanuele Menegatti, Francesco Piccione, José del R. Millán^*^

*** Correspondence:** Luca Tonin, [luca.tonin@epfl.ch](mailto:luca.tonin@epfl.ch)**,** José del R. Millán, [jose.millan@epfl.ch](mailto:jose.millan@epfl.ch)

# Supplementary Table 2

Table 2. Scores obtained by the SN patients on neuropsychological tests, with relative cut-off score for each test.

|  |  | Patient P1 | Patient P2 | Patient P3 |
| --- | --- | --- | --- | --- |
|  | **Cut-off** | **Score** | **Score** | **Score** |
| MMSE (Measso et al. 1993) | < 24 | 25,53 | 23,31*° | 25,62 |
| BIT-Conventional (Wilson et al. 1987) | < 130 | 104* | 122* | 131 |
| Bells test (Vallar et al. 1994) |  |  |  |  |
| Total omissions | ≤ - 5 / ≥ 5 | 2 | 4 | 3 |
| Omissions Left-Right | ≤ - 5 / ≥ 5 | 0 | 0 | 1 |
| Letter cancellation (Vallar et al. 1994) |  |  |  |  |
| Total omissions | ≤ - 5 / ≥ 5 | 1 | 3 | 2 |
| Omissions Left-Right | ≤ - 3 / ≥ 3 | -1 | 1 | 0 |
| Symbol cancellation (Vallar et al. 1994) |  |  |  |  |
| Total omissions | ≤ - 3 / ≥ 3 | 0 | 3* | 5* |
| Omissions Left-Right | ≤ - 3 / ≥ 3 | 0 | 3* | 3* |
| * Pathologic score. | | | | |
| ° Patient P2 was mainly impaired in the temporal judgment subtest. | | | | |
