# Supplementary material for: Behavioral and Cortical Effects during Attention Driven Brain-Computer Interface Operations in Spatial Neglect: A Feasibility Case Study
Source: Front Hum Neurosci. 2017 Jun 28;11:336. doi: 10.3389/fnhum.2017.00336 (PMC5487481; doi:10.3389/fnhum.2017.00336)
Supplement: Supplementary file 3 [file Table_3.DOCX]

Supplementary Material

Behavioral and Cortical Effects during Attention Driven Brain-Computer Interface Operations in Spatial Neglect:
A Feasibility Case Study

Luca Tonin^*^, Marco Pitteri, Robert Leeb, Huaijian Zhang, Emanuele Menegatti, Francesco Piccione, José del R. Millán^*^

*** Correspondence:** Luca Tonin, [luca.tonin@epfl.ch](mailto:luca.tonin@epfl.ch)**,** José del R. Millán, [jose.millan@epfl.ch](mailto:jose.millan@epfl.ch)

# Supplementary Table 3

Table 3. Number of runs and trials for each patient, modality and session. Between brackets the number of trials for the two experimental conditions (left attention task/rest).

|  | Patient 1 | | Patient 2 | | Patient 3 | |
| --- | --- | --- | --- | --- | --- | --- |
|  | **Calibration** | **Online** | **Calibration** | **Online** | **Calibration** | **Online** |
| Session 1 | 4 (40/40) |  | 2 (20/20) |  | 2 (20/20) |  |
| Session 2 | 1 (10/10) | 2 (40/20) | 2 (20/20) |  | 2 (20/20) |  |
| Session 3 |  | 3 (60/30) |  | 3 (60/30) |  | 4 (80/40) |
| Session 4 |  | 3 (60/30) |  | 3 (60/30) |  | 3 (60/30) |
| Session 5 |  | 3 (60/30) |  | 2 (40/20) |  | 3 (60/30) |
| Session 6 |  | 3 (60/30) |  | 2 (40/20) |  | 2 (40/20) |
| Total | 5 (50/50) | 14 (280/140) | 4 (40/40) | 10 (200/100) | 4 (40/40) | 12 (240/120) |
