# Supplementary material for: Behavioral and Cortical Effects during Attention Driven Brain-Computer Interface Operations in Spatial Neglect: A Feasibility Case Study
Source: Front Hum Neurosci. 2017 Jun 28;11:336. doi: 10.3389/fnhum.2017.00336 (PMC5487481; doi:10.3389/fnhum.2017.00336)
Supplement: Supplementary file 4 [file Table_4.DOCX]

Supplementary Material

Behavioral and Cortical Effects during Attention Driven Brain-Computer Interface Operations in Spatial Neglect:
A Feasibility Case Study

Luca Tonin^*^, Marco Pitteri, Robert Leeb, Huaijian Zhang, Emanuele Menegatti, Francesco Piccione, José del R. Millán^*^

*** Correspondence:** Luca Tonin, [luca.tonin@epfl.ch](mailto:luca.tonin@epfl.ch)**,** José del R. Millán, [jose.millan@epfl.ch](mailto:jose.millan@epfl.ch)

# Supplementary Table 4

Table 4. Definition of nodes for frontal, parietal and occipital regions and for each hemisphere.

| Region | Hemisphere | Channels |
| --- | --- | --- |
| *Frontal* | *Left* | F5, F3, F1, FC5, FC3, FC1, Fz, FCz |
| *Parietal* | *Left* | P7, P5, P3, P1, Pz |
| *Occipital* | *Left* | PO7, PO3, O1, POz, Oz |
| *Frontal* | *Right* | F6, F4, F2, FC6, FC4, FC2, Fz, FCz |
| *Parietal* | *Right* | P8, P6, P4, P2, Pz |
| *Occipital* | *Right* | PO8, PO4, O2, POz, Oz |
