# Supplementary material for: Behavioral and Cortical Effects during Attention Driven Brain-Computer Interface Operations in Spatial Neglect: A Feasibility Case Study
Source: Front Hum Neurosci. 2017 Jun 28;11:336. doi: 10.3389/fnhum.2017.00336 (PMC5487481; doi:10.3389/fnhum.2017.00336)
Supplement: Supplementary file 5 [file Table_5.DOCX]

Supplementary Material

Behavioral and Cortical Effects during Attention Driven Brain-Computer Interface Operations in Spatial Neglect:
A Feasibility Case Study

Luca Tonin^*^, Marco Pitteri, Robert Leeb, Huaijian Zhang, Emanuele Menegatti, Francesco Piccione, José del R. Millán^*^

*** Correspondence:** Luca Tonin, [luca.tonin@epfl.ch](mailto:luca.tonin@epfl.ch)**,** José del R. Millán, [jose.millan@epfl.ch](mailto:jose.millan@epfl.ch)

# Supplementary Table 5

Table 5. IAF [Hz] computed over modalities in parietal-occipital nodes belonging to each hemisphere. Mean and standard deviation are reported.

|  | **Calibration** | | **Online** | |  |
| --- | --- | --- | --- | --- | --- |
| Patient | *Left* | *Right* | *Left* | *Right* |  |
| P1 | 9.24±1.08 | 9.32±1.14 | 9.08±1.14 | 8.88±1.23 |  |
| P2 | 7.51±1.37 | 7.11±1.01 | 7.66±1.16 | 7.21±1.38 |  |
| P3 | 9.96±1.10 | 9.63±1.08 | 9.75±1.07 | 9.39±1.02 |  |
